# Striatal astrocytes engulf dopaminergic debris in Parkinson's disease: A study in an animal model

**DOI:** 10.1371/journal.pone.0185989

**Published:** 2017-10-13

**Authors:** Ingrid Morales, Alberto Sanchez, Clara Rodriguez-Sabate, Manuel Rodriguez

**Affiliations:** 1 Laboratory of Neurobiology and Experimental Neurology, Department of Basic Medical Sciences, Faculty of Medicine, Universidad de La Laguna, La Laguna, Tenerife, Canary Islands, Spain; 2 Center for Networked Biomedical Research in Neurodegenerative Diseases (CIBERNED), Madrid, Spain; University of Louisville, UNITED STATES

## Abstract

The role of astrocytes in Parkinson’s disease is still not well understood. This work studied the astrocytic response to the dopaminergic denervation. Rats were injected in the lateral ventricles with 6-hydroxydopamine (25μg), inducing a dopaminergic denervation of the striatum not accompanied by non-selective tissue damage. The dopaminergic debris were found within spheroids (free-spheroids) which retained some proteins of dopaminergic neurons (e.g., tyrosine hydroxylase, the dopamine transporter protein, and APP) but not others (e.g., α-synuclein). Free-spheroids showed the initial (LC3-autophagosomes) but not the late (Lamp1/Lamp2-lysosomes) components of autophagy (incomplete autophagy), preparing their autophagosomes for an external phagocytosis (accumulation of phosphatidylserine). Free-spheroids were penetrated by astrocyte processes (fenestrated-spheroids) which made them immunoreactive for GFAP and S100β, and which had some elements needed to continue the debris degradation (Lamp1/Lamp2). Finally, proteins normally found in neurons (TH, DAT and α-synuclein) were observed within astrocytes 2–5 days after the dopaminergic degeneration, suggesting that the intracellular contents of degenerated cells had been transferred to astrocytes. Taken together, present data suggest phagocytosis as a physiological role of striatal astrocytes, a role which could be critical for cleaning striatal debris during the initial stages of Parkinson’s disease.

## Introduction

There is increasing evidence suggesting that the degeneration of dopamine neurons (DA-ergic neurons) which characterizes Parkinson’s disease (PD) starts in the striatal synapse and progresses by dying-back degeneration of the axon to the cell soma in the substantia nigra (SN) [1]. Dopamine (DA), tyrosine hydroxylase (TH) and DA membrane transporter (DAT) present a more marked decrease in the striatum than in the SN, a fact observed during the first stages of the illness but also in patients with a medium-term or long-term evolution [2]. Under normal conditions, DA-ergic neurons present a slow degeneration with aging (6–8% of cells every decade), a fact that induces PD when more than 60% of the striatal synapses have been lost. Thus, the synaptic debris produced by the aged-related or parkinsonian degeneration of DA-ergic neurons needs to be continuously withdrawn from the striatal tissue, which could be performed by the macroautophagy (hereafter referred to as autophagy), microautophagy and chaperone-mediated autophagy [3–5] of DA-ergic neurons, or by microglial phagocytosis [6]. Microglia and macrophages are the “professional” phagocytes in the brain, but their activation facilitates a release of cytokines, chemokines and ROS which could accelerates the DA-ergic neuron degeneration [7, 8]. Astrocytes also express components of different phagocytic pathways [9], which made them a possible alternative for removing DAergic debris (e.g., for removing the α-synuclein accumulated in PD) [10–13]. Striatal astrocytes have been involved in the starting and progression of PD but, because astrocytes may increase [8, 11, 14] and prevent [15–18] the neuronal damage, their actual role in the DA-ergic neuron degeneration is controversial [19].

The present work was aimed at studying the possible phagocytic activity of astrocytes as mechanisms for cleaning the DAergic debris produced by the degeneration of the striatal DAergic terminals. The initial hypothesis was that the phagocytic activity of astrocytes is enough to keep the striatum free of DAergic debris, thus preventing the recruitment of microglia and the cytotoxic effects of these cells on DA-ergic neurons. The 6-hydroxydopamine (6OHDA) injection in the striatal tissue has proved useful to degenerate the synaptic terminals of DA-ergic neurons. However, this procedure is normally accompanied by a microglial activation [20, 21] generated by the unspecific damage of tissue surrounding the injection locus (e.g., needle penetration, high hydrostatic pressure generated by the injection, high 6OHDA-concentration around the needle tip…) [22]. Thus, a recently reported modification of this method (based on injecting 6OHDA in the lateral ventricle) which proved suitable for producing a striatal DAergic denervation without inducing non-selective tissue damage was used [23, 24].

## Methods

### Animals and lesions

Experiments were carried out on 45 male Sprague-Dawley rats weighing 300–350 g. Animals were housed at 22°C, two per cage, under normal laboratory conditions on a standard light-dark schedule with free access to food and water. Experiments were conducted in accordance with the European Communities Council Directive of 24 November 1986 (86/609/EEC) regarding the care and use of animals for experimental procedures and adequate measures were taken to minimise pain and discomfort. All studies were approved by the La Laguna University Animal Care and Use Committee.

Rats were anaesthetised with ketamine (25–40 mg/kg i.p.; Rhône Mérieux; Lyon, France) and xylazine (3–6 mg/kg i.p.; Bayer, Leverkusen, Germany), and injected in the lateral ventricle (Kopf Instruments, Tujunga, California; coordinates: 1.4 mm lateral to the midline, 0.8 mm posterior to bregma and 4 mm below the dura) with vehicle (0.9% saline solution with 0.3 μg/μl ascorbic acid) or a single dose of 6OHDA (6-hydroxydopamine hydrochloride, Sigma, St. Louis, MO, 25μg in 10 μl of vehicle per injection; 1 μl/min). In order to prevent the degeneration of noradrenergic cells, the noradrenaline uptake was inhibited with nortriptyline (30 mg/kg injected i.p. 20 min before 6-OHDA administration; nortriptyline hydrochloride, Sigma, St. Louis, MO).

### Tissue processing

Rats were anaesthetized with chloral hydrate (400 mg/kg i.p.) and, after checking for complete anaesthetic state (e.g. loss of corneal reflex by lack of blinking when air is blown into eyes and loss of pedal pain reflex by lack of movement of paw/tail when squeezed), they were transcardially perfused with 200 ml of 0.9% saline solution followed by 400 ml of 4% paraformaldehyde in 0.1 M phosphate buffer pH = 7.4 (PBS) 4 hours, 1 day and 5 days after 6-OHDA administration, [15]. Brains were removed and stored in the same fixative at 4°C for 4 hours, immersed in a cryoprotective solution of 30% sucrose in the same buffer for 48 hours and then cut at 30 μm with a sliding microtome (HM 450, MICROM International GmbH; Walldorf). Brains were cut following axial planes parallel to the surface of the brain cortex and perpendicular to the probe trajectory. Sections were collected in 7 parallel series and processed for immunohistochemistry [25].

### Immunofluorescent labelling

For immunofluorescent labeling, floating sections were first incubated for 1 hour at room temperature (RT) in 4% normal goat serum (NGS, Sigma-Aldrich, Madrid) in PBS and 0.05% Triton X-100 (TX-100, Sigma-Aldrich, Madrid), and overnight in the same solution containing the primary antibodies shown in Supplementary Information. Finally, triple and quadruple immunofluorescent labeling sections were incubated for 2 hours with the secondary antibodies (Table 1) in PBS containing 1:200 NGS followed by CY2, CY3, DyLight 649 conjugated streptavidine (Jackson ImmunoResearch) in PBS.

**Table 1:** Antibodies used in the study.

**Primary Antibodies**	**Dilution**	**Host**	**Producer (Catalogue number)**
TH	1:1200	chicken	Abcam, ab76442
DAT	1:50	goat	Santa Cruz Biotechnology,Inc.,sc1433
GFAP	1:1000	mouse	Millipore, MAB360
GFAP	1:600	rabbit	Sigma, G9269
GS	1:1000	rabbit	Abcam, ab93439
S100β	1:400	rabbit	LSBio, LS-B2601
APP	1:1500	rabbit	Abcam, ab32136
SYN	1:200	rabbit	Sigma, S3062
LC3-II	1:1200	rabbit	Abcam, ab48394
LC3-II	1:200	goat	Santa Cruz Biotechnology,Inc., sc16755
LAMP1	1:100	mouse	Abcam, ab25630
LAMP2	1:100	goat	Santa Cruz Biotechnology,Inc., sc8100
PS (AF 548 Annexin V)	1:20	-	Invitrogen, A13202
Stx17	1:200	rabbit	ProteinTech, 17815-1-AP
Ubi	1:250	rabbit	NovusBio, NB100-92119
**Secondary Antibodies**	** **	** **	** **
AF 488 anti-Chicken	1:400	Donkey	Jackson ImmunoResearch, 703-545-155
AF 647 anti-Chicken	1:400	Donkey	Jackson ImmunoResearch, 703-605-155
Cy3 anti-Goat	1:400	Donkey	Jackson ImmunoResearch, 705-165-147
AF 488 anti-Goat	1:400	Donkey	Jackson ImmunoResearch, 705-545-147
AF 647 anti-Mouse	1:400	Donkey	Jackson ImmunoResearch, 715-605-151
Biotin anti-Mouse	1:300	Donkey	Jackson ImmunoResearch, 715-065-151
Cy3 anti-Rabbit	1:400	Donkey	Jackson ImmunoResearch, 711-165-152
Biotin anti-Rabbit	1:1000	Donkey	Jackson ImmunoResearch, 711-065-152
Biotin anti-Sheep	1:600	Donkey	Jackson ImmunoResearch, 713-065-147
AF 647 anti-Chicken	1:500	Goat	Jackson ImmunoResearch, 103-605-155
AF 488 anti-Chicken	1:800	Goat	Invitrogen, A11039
AF 546 anti-Chicken	1:600	Goat	Invitrogen, A11040
AF 546 anti-Mouse	1:1000	Goat	Invitrogen, A21123
FITC anti-Mouse	1:400	Goat	Jackson ImmunoResearch, 115-095-003
Biotin anti-Mouse	1:1200	Goat	Jackson ImmunoResearch, 115-065-003
Cy3 anti-Rabbit	1:500	Goat	Jackson ImmunoResearch, 111-165-144
Biotin anti-rabbit	1:1200	Goat	Jackson ImmunoResearch, 111-065-144
FITC anti-Rabbit	1:100	Goat	Jackson ImmunoResearch, 111-095-045
**Others**			
405-streptavidin	1:400	-	Jackson ImmunoResearch, 016-470-084
Dylight 649-streptavidin	1:600	-	Jackson ImmunoResearch, 016-490-084
488-streptavidin	1:800	-	Jackson ImmunoResearch, 016-540-084
Cy3-streptavidin	1:600	-	Jackson ImmunoResearch, 016-160-084

After several rinses, sections were mounted on gelatinized slides, air dried, coverslipped with Vectashield (Vector), and examined under confocal microscopy (Olympus Fluoview FV1000 and Leica SP8) using appropriate filters. Appropriate controls were performed to test the immunohistochemical assays. Primary antibodies were selected because they had been previously tested in different studies and in many brain sites including the striatum, and because the selectivity for their biomolecular targets was confirmed by western blot and absorption controls. Before applying primary antibodies, tissues were inspected under bright-field and fluorescence microscope to ensure that the endogenous tissue background was not interfering with the study. Controls with the tissue incubated without the primary antibody but including the secondary antibody were also performed. When necessary, control studies were also performed where the primary antibody was replaced by a specific isotype (Chicken IgY isotype control, AB-101-C, Novus Biologicals USA; Goat IgG isotype control, AB-108-C, Novus Biologicals USA; Rabbit IgG monoclonal [EPR25A] isotype control, ab 172730, Abcam, UK; Mouse IgG1 isotype control, NBP1-97005, Novus Biologicals USA; Rabbit IgG isotype control, NBP2-21952, Novus Biologicals USA). During the initial optimization studies, all antibodies were tested in tissue samples or cells that are known to express the epitope of interest. These control tests were performed during the initial optimization studies and, when possible, they were also performed in the same tissue samples (looking for other cells or sub-cellular components which are known to present the epitope of interest). Quantitative analysis was performed with the ImageJ (IJ1.46r) software.

### Tracing study

Four rats anaesthetized with ketamine/xylazine received a single injection of the biotinylated dextran amine (BDA; 1 μg in 0.3 μl; 0.3 μl/min) in the posterior region of the medial forebrain bundle (4 mm posterior to Bregma, 1 mm lateral to the mid-line and 7 mm ventral to cortical surface). One month later, rats were i.p. injected with nortriptyline (30 mg/kg) and 20 min later with a single dose of 6OHDA (25μg in 10 μl administered in the lateral ventricle; 1 μl/min). Three (2 rats) and five (2 rats) days after 6OHDA administration, the rats were anaesthetized with chloral hydrate (400 mg/kg i.p.) and transcardially perfused with 200 ml of 0.9% saline solution followed by 400 ml of 4% paraformaldehyde in 0.1 M PBS. Finally, the brains were removed and stored in the same fixative (4°C for 4 hours) immersed in 30% sucrose in the same buffer (4°C for 40 hours) and then cut at 30 μm with the sliding microtome.

### Quantitative analysis and statistical comparisons

The diameter of the spheroids was computed in striatal areas ipsilateral to the 6OHDA administration and which showed a full dopaminergic denervation. The diameter of synapses was computed in the striatum contralateral to 6OHDA administration and which, as previously reported [23, 24], showed no dopaminergic denervation. The immunoreactivity of proteins was evaluated by a densitometric analysis of DAergic spheroids, astrocytic processes and astrocytic soma. Densitometric data were normalized as a percentage of the mean value found in the extracellular locations or in the synaptic boutons of the contralateral striatum. In order to prevent differences due to variations in protocol conditions during tissue processing and densitometric analysis, all sections were processed simultaneously using the same protocols and chemical reagents, and all microscopic and computer parameters were kept constant throughout the densitometric study. Measurements were performed with the FV10-ASW (version 01.07.01.00; Olympus Corporation) and ImageJ (IJ 1.46r) software. Subcellular co-localizations were performed with the Leica Application Suite Advanced Fluorescence, and with the Colocalization, RG2B colocalization, Colocalization_Finder and JACoP plugins of ImageJ program [26, 27]. Colocalizations were quantified with Pearson’s correlation coefficient, the cytofluorogram, the Manders overlap coefficients M1 (fraction of the marker 1 overlapping with marker 2) and M2 (fraction of the marker 2 overlapping with marker 1), the co-localization rate (the extent of colozalization in percentages), and the overlap coefficient (a value between 0 and 1 -the closer the value is to 1, the stronger the colocalization-).

Statistical analyses were performed using the Scheffé test for sample comparisons (statistic program Statsoft; Tulsa, U.S.A.; p<0.001 was considered as critical for assigning statistical significance).

## Results

### The adaptive reaction of DAergic terminals to damage: Forming free-spheroids

**Fig 1:**
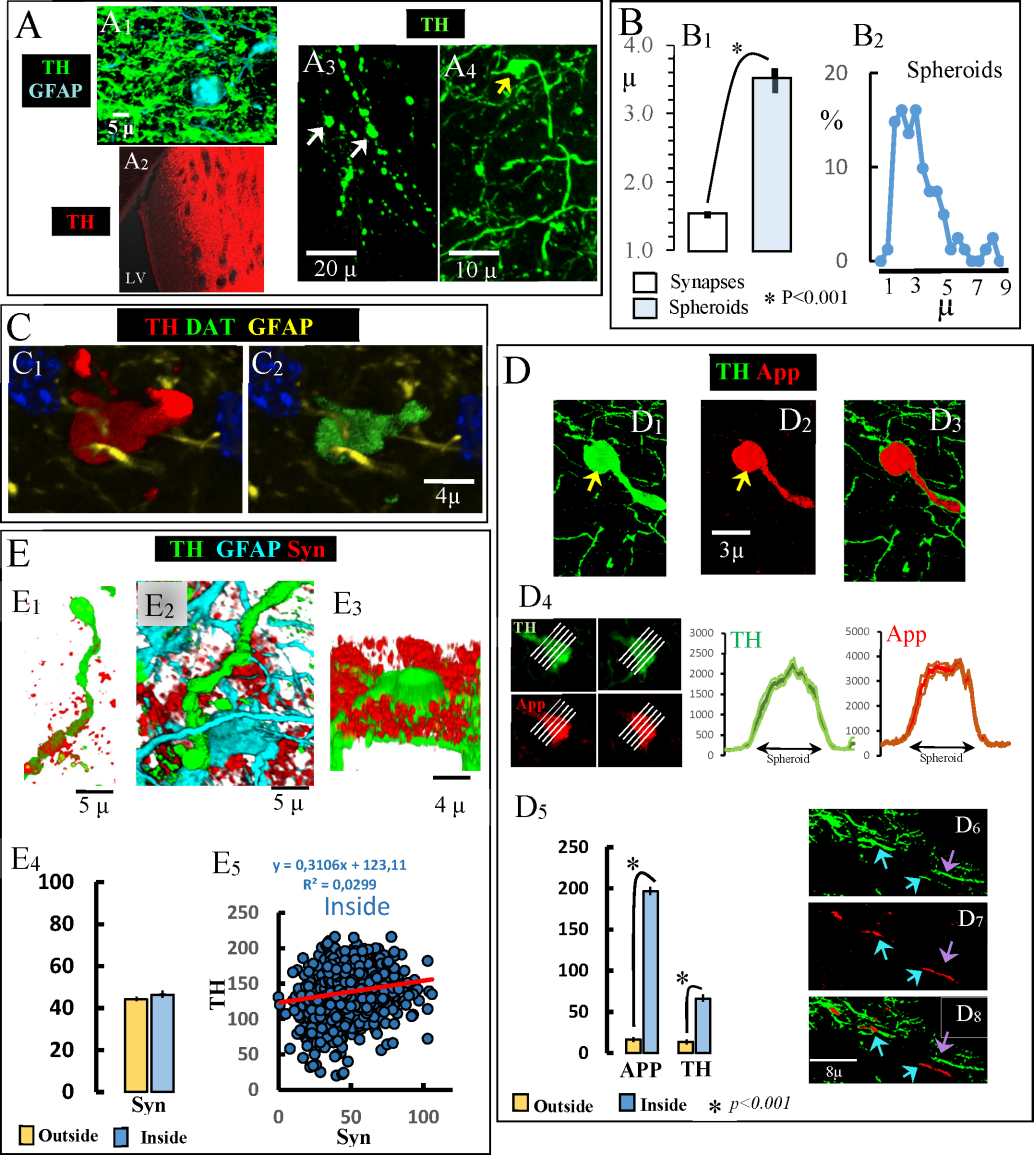
Accumulation of dopaminergic debris in end-bulb-like structures and free-spheroids. A1: dopaminergic terminal in close proximity to a striatal astrocyte. A2: The 6OHDA perfusion in the lateral ventricle degenerated the DAergic innervation in striatal regions near the ventricle. A3: white arrows show two spheroids generated by the fragmentation of DAergic axons. A4: yellow arrow shows an end-bulb-like structure at the end of a degenerating DAergic axon. B1: diameter of free-spheroids vs. DAergic synapses (mean ± standard error). B2: distribution of the diameter of free-spheroids (n = 400). C: an example of a spheroid accumulating TH (C1) and DAT (C2). D: show examples of App accumulation (D3) in an end-bulb-like structure (yellow arrow in E1-E2). D4 shows the TH and App distribution (right-side) of a spheroid which showed App (left-side) (distributions represent the mean ± standard error of immunoreactivity values across the lines shown in the left-side images). D5: mean ± standard error of the immunoreactivity values computed inside vs. outside the spheroids. D6-D8 App in degenerating TH-axons (blue arrows showing both App and TH immunoreactivity and the violet arrow showing TH but not App immunoreactivity). E1-E3 show three examples of Syn accumulation in the extracellular space near degenerating DAergic axons. E4 shows the mean ± standard error of the Syn immunoreactivity values computed inside vs. outside the spheroids. E5 shows no TH-Syn co-localization inside spheroids.

There is a massive DAergic innervation of the striatum whose terminals are in close proximity to striatal astrocytes (Panel A1 in Fig 1). The 6OHDA perfusion in the lateral ventricle degenerated DAergic axons and synaptic terminals in the ipsilateral striatum near the ventricle (Panel A2 in Fig 1). The DAergic degeneration generated debris within fragmented axons which formed end-bulb-like structures at the end of degenerating axons (yellow arrow in Panel A4 in Fig 1) and spheroids (white arrows in Panel A3 in Fig 1) free of connections to astrocytes (S1 Animation) and which will be referred to here as **free-spheroids**. Free-spheroids had a diameter of around 4 microns (Panel B1 in Fig 1 shows the diameter of spheroids identified in the fully denervated striatum and of synapses of the contralateral non-denervated striatum), with a diameter in the range between 1 and 9 microns (Panel B2 in Fig 1). Free-spheroids showed proteins which are normally found in DA-ergic neurons, including TH (Panel C1 in Fig 1) and DAT (Panel C2 in Fig 1). The amyloid precursor protein (App; a protein normally found in synapses) was found in end-bulb-like structures and free-spheroids (Panels D1-D3 in Fig 1) and in some (blue arrows in Panels D6-D8 in Fig 1) but not all (violet arrows in Panels D6-D8 in Fig 1) damaged axons. Left side images in Panel D4 in Fig 1 show an example of two slices obtained from a spheroid and used to compute the distribution of TH and App immunoreactivity across the spheroid (this computation was performed for each of the white lines in the figures). As shown on the right-side of Panel D4 in Fig 1 (which represents the mean ± standard error of the ten lines used for each protein), both the TH and App were distributed across the spheroid. Thus, the immunoreactivity inside the spheroid was higher than that found in the extracellular space outside the spheroid (Panel D5 in Fig 1). Other synaptic proteins such as α-synuclein (Syn) were not found in either free-spheroids or in fragmented axons or end-bulb-like structures. Although Syn immunoreactivity was some time found in the extracellular space near fragmented axons (Panels E1-E3 in Fig 1), no statistical difference was found for Syn immunoreactivity inside vs. outside the spheroid (Panel E4 in Fig 1), and no correlation was found inside the spheroid between the immunoreactivity of Syn and TH (Panel E5 in Fig 1). Thus, proteins of degenerating DAergic terminals were found within free-spheroids, a fact that did not occur with Syn.

### The metabolization of proteins in free-spheroids

**Fig 2:**
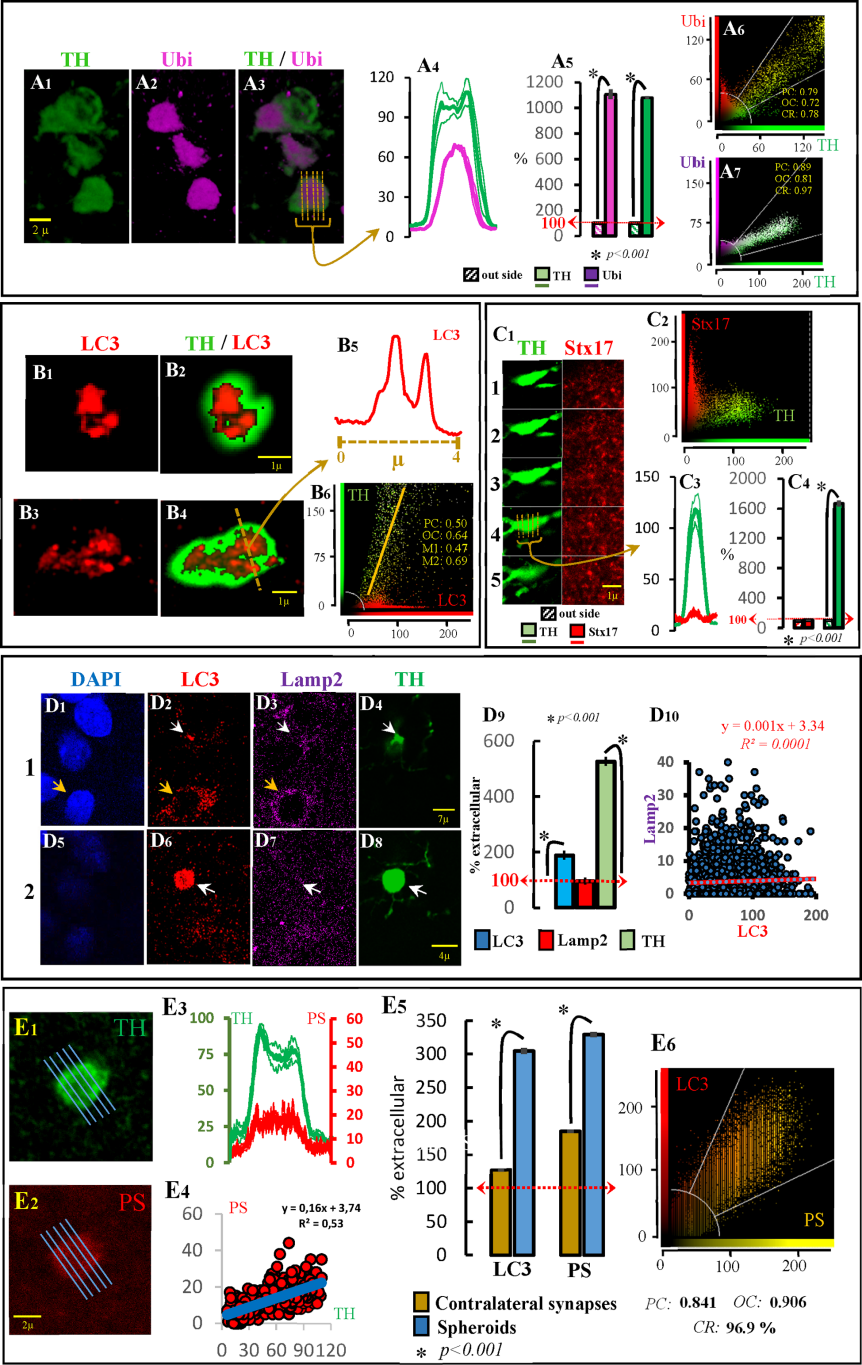
Free-spheroids and autophagy. The ubiquitin (Ubi) immunoreactivity in spheroids is shown in the A figures. A1 shows three spheroids with marked Ubi immunoreactivity (A2, A3). The distribution of Ubi immunoreactivity in one of these spheroids is shown in A4. Thus, the Ubi and TH immunoreactivity computed in spheroids (n = 200) vs. those recorded in the extracellular space is shown in A5. A6 and A7 show two characteristic examples of Ubi-TH co-localization computed in the denervated striatum. The LC3 immunoreactivity in spheroids is shown in the B figures. B1 and B3 show two examples of LC3 immunoreactivity in TH+ spheroids (B2 and B4 respectively). The distribution of LC3 immunoreactivity across the line indicated in B4 is shown in B5 (vesicular distribution). B6 shows the LC3-TH co-localization computed in the denervated striatum. The syntaxin-17 (Stx17) immunoreactivity in spheroids is shown in the C figures. C1 shows five consecutive slices of a TH+ spheroid with no aggregation of Stx17. C2 shows no LC3-TH co-localization computed in the denervated striatum. C3 shows the Stx17 and TH distribution across slice 4 in fig C1. C4 shows the Stx17 and TH immunoreactivity computed in spheroids (n = 200) vs. those recorded in the extracellular space. The LC3 and Lamp2 immunoreactivity in TH+ spheroids is shown in the D figures. D1-D4 and D5-D8 show two consecutive slices with a TH+ spheroid (D4 and D8; white arrow) showing LC3 (D2 and D6) but not Lamp2 (D3 and D7) immunoreactivity. The distribution of both markers in the soma of a striatal cell (useful to verify that the Lamp2 antibody was working) is indicated with a yellow arrow in D1-D3. The LC3, Lamp2 and TH immunoreactivity computed in spheroids (n = 200) vs. those recorded in the extracellular space is shown in D9. There was no LC3-Lamp2 co-localization in spheroids (D10).The phosphatidylserine (PS) immunoreactivity in spheroids is shown in the E figures. An example of a TH+ spheroid showing a marked PS immunoreactivity is shown in E1 and E2 respectively. The distribution and the co-localization of TH and PS immunoreactivity distribution across this spheroid is shown in E3 and E4 respectively. The LC3 and TH immunoreactivity computed in spheroids (n = 200) vs. those recorded in the extracellular space is shown in E5. The LC3-PS co-localization within spheroids (n = 25) is shown in E6. A4, A5, C3, C4, D9, E3 and E5 show the mean ± standard error. PC: Pearson’s correlation; OC: overlap coefficient; CR: co-localization rate.

The immunohistochemical detection of ubiquitin is commonly used to detect pathological accumulations of proteins in cell compartments. The polyclonal antibody used here (which has a high affinity for ubiquitin-protein conjugates compared with free ubiquitin) showed a marked immunoreactivity for ubiquitin in free-spheroids (Panels A1-A3 in Fig 2). The ubiquitin immunoreactivity was 1000% higher in spheroids than in the surrounding striatal regions (Panels A4 and A5 in Fig 2). Ubiquitin also showed a notable co-localization with TH in the denervated striatum where practically all TH immunoreactivity was located in spheroids (see two examples in Panels A6 and A7 in Fig 2, and S2 Animation).

The immunohistochemical detection of LC3 (microtubule-associated protein 1 light chain 3 which is the mammalian homolog of yeast Atg8) aggregates is commonly used to detect the generation of autophagosomes. There are two LC3 proteins, LC3-I normally located in the cytosol (where it presents a low concentration and low immunoreactivity) and LC3-II which is accumulated in the autophagosome membranes. There are no selective antibodies for LC3-II (the LC3 antibody used here recognizes both the LC3-I and LC3-II), and the finding of high LC3 immunoreactivity distributed in small aggregations (LC3 puncta) is necessary to associate the LC3 immunoreactivity to the generation of autophagosomes (the finding of LC3-immunoreactivity dispersed across the cytosol is not enough). A high LC3-immunoreactivity in spheroids (see in Panel B6 in Fig 2 the LC3-TH co-localization found in denervated areas of the striatum) and a vesicular aggregation of the LC3 immunoreactivity within spheroids (see two examples in Panels B1 vs. B2 and B3 vs. B4 in Fig 2) were both observed here. These aggregations correspond with the LC3 puncta which may be observed with light microscopy in the soma of cells producing autophagosomes, but whose identification in the small volumes of spheroids requires high-magnification images such as those obtained here by confocal methods (Panel B5 in Fig 2). These findings suggest that TH+ spheroids generate autophagosomes.

Syntaxin-17 (Stx17) is the SNARE protein of autophagosomes. Stx17 is normally present in the cytosol from where it might be recruited by mature autophagosomes (it only appears in late autophagosomes with sealed membranes and not in phagophores and early autophagosomes). Stx17 is necessary for the transport of autophagosomes to the cell soma (by dynein motors) and for the fusion of autophagosomes to lysosomes. Stx17 was not observed in the autophagosomes of free-spheroids. Fig 2C1shows an example of five consecutive slices of a TH+ spheroid showing no aggregation of Stx17. Stx17 immunoreactivity did not increase across (Panel C3 in Fig 2) or within (Panel C4 in Fig 2) spheroids. In addition, no TH-Stx17 co-localization was found in the denervated striatal tissue (Panel C2 in Fig 2).

The immunohistochemical detection of Lamp1 and Lamp2 are commonly used as markers of lysosomes. The example shown in Panels D1-D8 in Fig 2 presents two consecutive striatal slices where both LC3 (Panel D2 in Fig 2) and Lamp2 (Panel D3 in Fig 2) can be observed in cytosolic regions near the nucleus of cells (yellow arrows), and where TH+ spheroid showed LC3 but not Lamp2 immunoreactivity (white arrows in Panels D2-D4 in Fig 2 and in Panels D6-D8 in Fig 2). LC3 immunoreactivity was higher (200%) in spheroids than in the extracellular space, a fact which was not observed for Lamp2 (Panel D9 in Fig 2). In agreement, no LC3-Lamp2 co-localization was found in spheroids (Panel D10 in Fig 2), suggesting that the production of autophagosomes in TH+ spheroids was not followed by the generation of autophagolysosomes.

Taken together, these findings suggest that spheroids accumulate ubiquitinated proteins and generate autophagosomes, thus starting the autophagy process. However, the autophagy process does not finish in the free-spheroid where the autophagosome does not mature (incorporating the Stx17 protein) and does not merge with the lysosome to finish the degradation of its content. Thus, the question was how the DAergic debris found in spheroids can be metabolized and eliminated.

Phosphatidylserine (PS) is a phospholipid normally located in the internal portion of the cell membrane and which is moved to the external portion of cell membranes under stressing conditions (where it acts as an “eat-me” signalling for phagocytosis). PS was found in spheroids, but not in their external membrane. Instead, PS was found in intra-cytosolic regions (Panels E1 and E2 in Fig 2) clearly located inside spheroids (Panel E3 in Fig 2), showing a TH-PS co-localization in striatal regions with a marked DAergic denervation and where most TH immunoreactivity was located in spheroids (Panel E4 in Fig 2) (see also S3 and S4 Animations). PS and LC3 observed within spheroids (Panel E5 in Fig 2 shows higher immunoreactivity values in spheroids of the denervated striatum than in synaptic structures of the un-lesioned contralateral striatum) showed co-localization (Panel E6 in Fig 2), suggesting that PS was present in the autophagosomes of spheroids.

On the whole, these data suggest that degenerating terminals of DA-ergic neurons present an autophagocytic process which begins (by making autophagosomes), but does not finish (lack of Stx17 and Lamp2) the degradation of DAergic debris. The signalling of cytosolic aggregates with PS and the progressive approximation of astrocyte processes to DAergic spheroids observed the days following the 6OHDA administration (see next paragraph) suggested the involvement of astrocytes in the processing of DAergic debris.

### The initial reaction of astrocytes to DAergic damage: Forming fenestrated-spheroids

**Fig 3:**
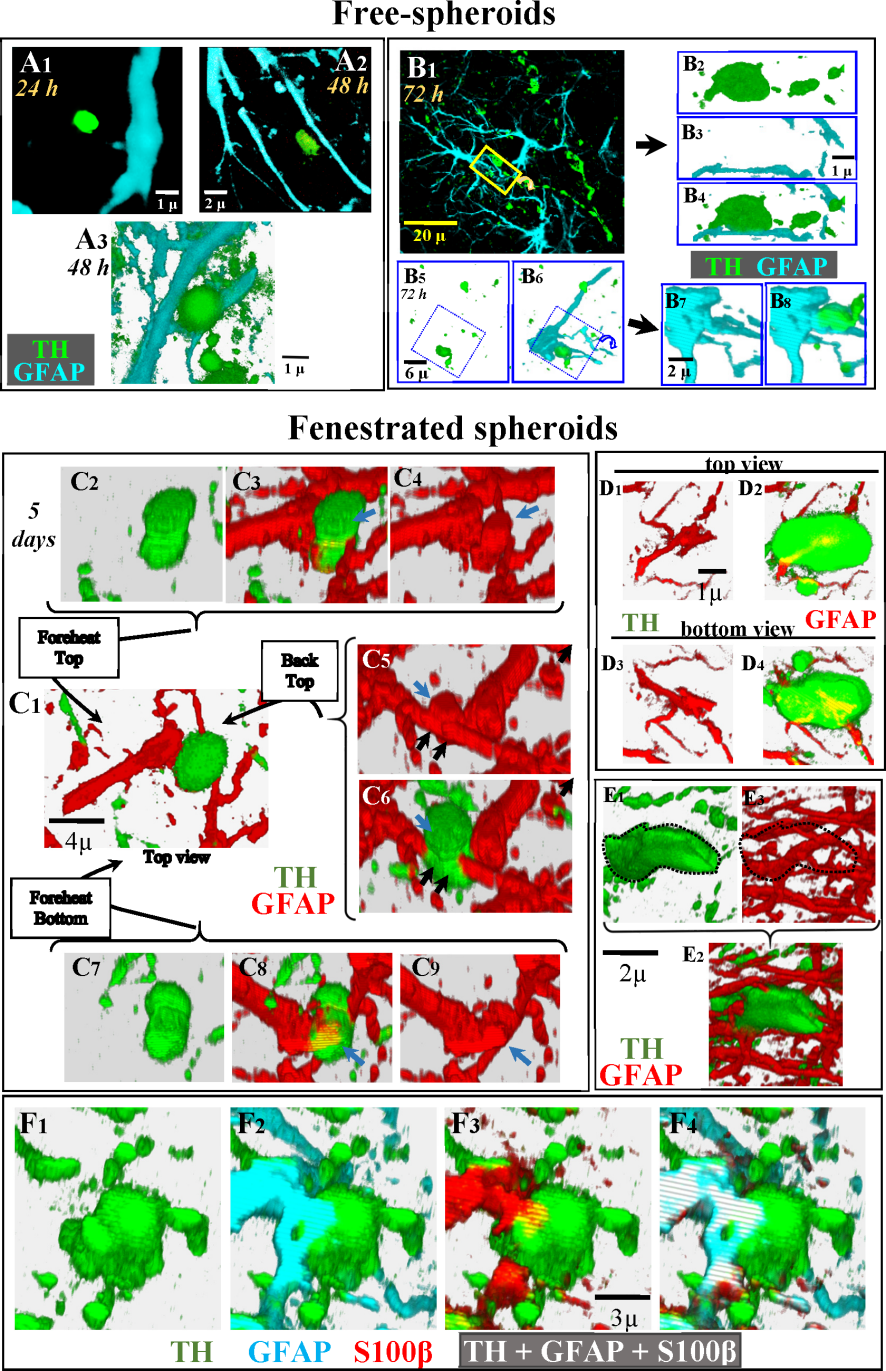
Identification of fenestrated-spheroids. Figures in A show examples of the position of DAergic spheroids with respect to astrocytic processes 24 (A1) and 48 (A2 and A3) hours after 6OHDA administration. The figures in B show two examples of the position of DAergic spheroids with respect to astrocytic processes 72 hours after 6OHDA administration. B2-B4 show an amplification and rotation of the yellow square shown in B1. B7 and B8 show an amplification and rotation of the blue square shown in B5-B6. Images C2-C9 present different perspectives of the spatial relationship between a spheroid (green) and a process (red) of an astrocyte (C1), showing that the spheroid is crossed by the astrocytic processes (black arrows in C5 and C6) and that the astrocytic crossing process emits an additional process inside the spheroid which progresses longitudinally across the largest diameter of the spheroid (blue arrows in C3 vs. C4, C5 vs. C6 and C8 vs. C9). D1-D4 images show the top (D1, D2) and bottom (D3, D4) perspectives of a spheroid penetrated by an astrocytic process which ramified into two branches inside the spheroid. A spheroid penetrated by multiple astrocytic processes is shown in E1-E3 (the spheroid boundary is drawn with a dotted line in E1 and E3). Figs F1-F4 present a fenestrated spheroid where the co-localization of GFAP, and S100β inside a TH-immunoreactive spheroid is shown.

A1 in Fig 3 shows a typical example of the position of spheroids regarding the astrocytic processes during the first 24 hours after 6OHDA administration. After 48 hours the distance between astrocytic processes and spheroids seems to decrease (Panels A2 and A3 in Fig 3). Panels B1-B8 in Fig 3 present two examples (with the respective magnifications) of the spatial relationship between spheroids and the soma (Panels B5-B8 in Fig 3) or processes (Panels B1-B4 in Fig 3) of astrocytes 72 hours after 6OHDA administration. Five days after 6OHDA, free-spheroids were surrounded and penetrated by astrocytic processes. The 3D-images in Panel C2-C9 in Fig 3 show the spatial relationship between a typical DAergic spheroid and the processes of surrounding astrocytes from different perspectives (Panel C1 in Fig 3). In this representative example, the spheroid is transversally crossed by an astrocyte process (black arrows in Panels C5 and C6 in Fig 3) which emits a transversal process inside the spheroid which progresses longitudinally across the largest diameter of the spheroid (see also blue arrows in Panels C3-C4 in Figs 3 and Panels C8-C9 in Fig 3). Panels D1-D4 in Fig 3 show the top (Panels D1 and D2 in Fig 3) and bottom (Panels D3 and D4 in Fig 3) views of an astrocytic process which ramified into two branches inside a spheroid. Some spheroids were penetrated by many processes coming from different astrocytes, an example of which is shown in images Panel E1-E3 in Fig 3 (the spheroid boundary is drawn with a dotted line in panels E1 and E3 in Fig 3). Spheroids penetrated by astrocytic processes will be referred to here as **fenestrated-spheroids** (see also S5 Animation where the spheroid is shown in green -TH- and the astrocytic process in red -GFAP-). Fenestrated-spheroids were identified in 2D-images (Panel F1 in Fig 3) when they present TH/GFAP (Panel F2 in Fig 3), TH/S100β (Panel F3 in Fig 3) or TH/GFAP/S100β (Panel F4 in Fig 3) immunoreactivity.

**Fig 4:**
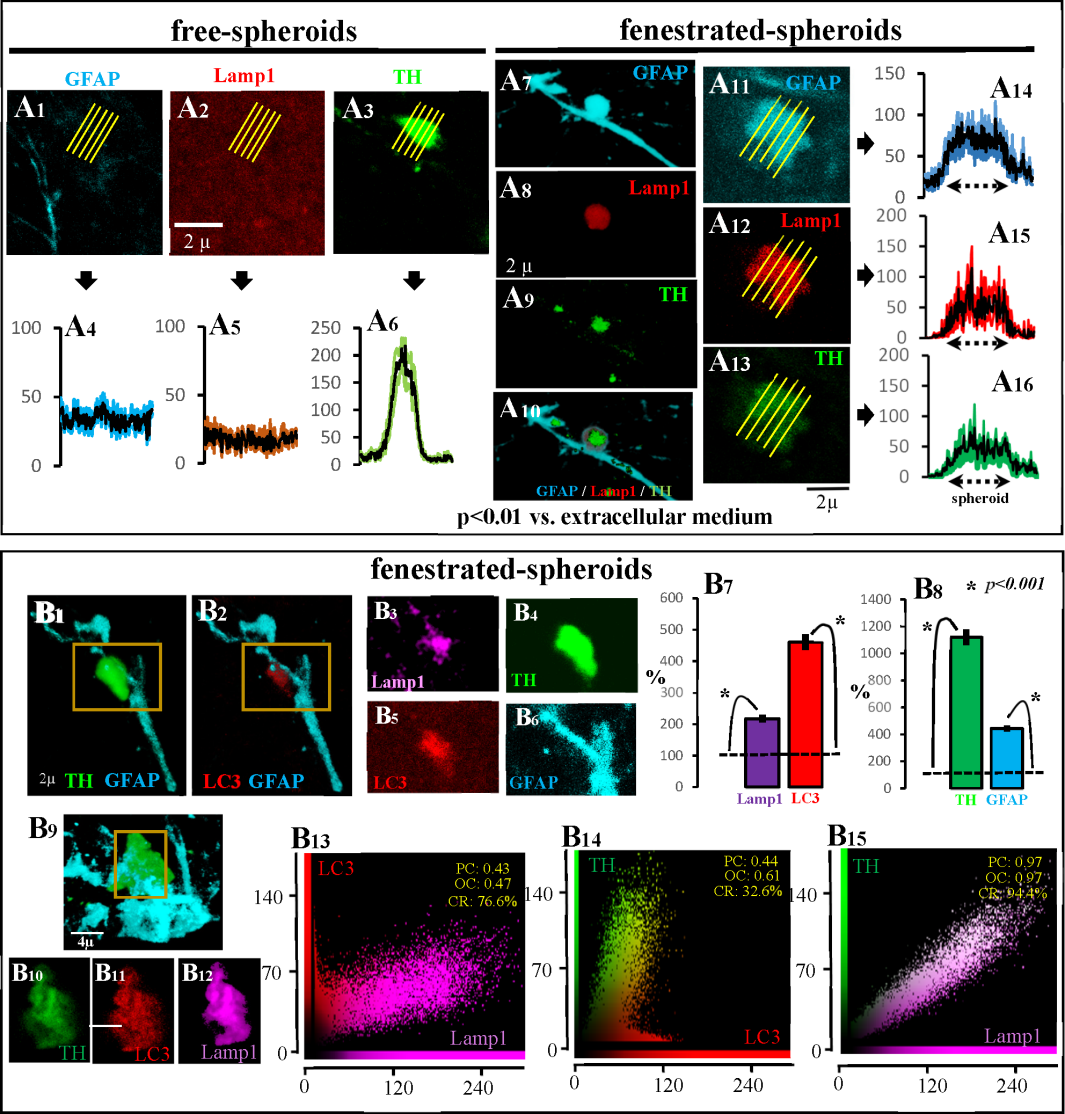
Main characteristics of fenestrated-spheroids. A1-A6 show an example of the absence of Lamp1 (A2) in free-spheroids which present TH (A3 and A6) but not GFAP (A1 and A4) immunoreactivity. Figs A7-A10 show an example of a fenestrated-spheroid (A10) showing TH (A9), GFAP (A7) and Lamp1 (A8) immunoreactivity (this example is also shown in S6–S9 Animations). Figs A11-A16 show another example of a fenestrated-spheroid showing TH (A13), GFAP (A11) and Lamp1 (A12) immunoreactivity (the immunoreactivity distribution within spheroids is shown in A16, A14, and A15, respectively). The fenestrated-spheroids (B4/B6 present an amplification of the yellow square of B1) which showed high LC3 immunoreactivity (B2/B5) also showed the lysosomal marker Lamp1 (B3). Thus, LC3 and Lamp1 immunoreactivity (B7) was higher in fenestrated spheroids (B8) than in the surrounding extracellular space (B7 and B8 show the mean ± standard error of values normalized according to the extracellular immunoreactivity). B10-B12 show an amplification of the yellow square shown in B9. The LC3 (B11) and Lamp1 (B12) observed in fenestrated-spheroids (B9/B10) showed co-localization inside the spheroid (B13; PC: Pearson’s correlation; OC: overlap coefficient; CR: co-localization rate), suggesting the production of autophagolysosomes in the fenestrated-spheroids. The findings of TH-LC3 (B14) and of TH-Lamp1 (B15) co-localization suggest that DAergic debris is being stored in autophagolysosomes.

As previously commented, free-spheroids showed no immunoreactivity for the lysosome marker Lamp2 (Panels D1-D10 in Fig 2). The same fact was found when Lamp1 was used as lysosome marker. The top of Fig 4 shows an example of a free-spheroid (with TH-immunoreactivity -Panels A3 and A6 in Fig 4- but not with GFAP-immunoreactivity -Panels A1 and A4 in Fig 4-) showing no Lamp1 immunoreactivity (Panels A2 and A5 in Fig 4). However, this fact changed in fenestrated-spheroids which showed Lamp1 immunoreactivity (Panels A12 and A15 in Fig 4), together with TH (Panels A13 and A16 in Fig 4) and GFAP (Panels A11 and A14 in Fig 4) immunoreactivity. Another example is shown in Panels A7-A10 in Fig 4 (this example can also be observed in S6, S7, S8 and S9 Animations where TH is shown in green, GFAP in blue and Lamp1 in red). Fenestrated-spheroids with high LC3 immunoreactivity (Panels B1 and B2 in Fig 4 and Panels B4-B6 in Fig 4), also showed a high level of the lysosomal marker Lamp1 (Panel B3 in Fig 4), a fact which was verified when the immunoreactivity of LC3 and Lamp1 (Pabel B7 in Fig 4) of fenestrated spheroids (Panel B8 in Fig 4) were compared with the immunoreactivity of the surrounding extracellular space (normalized to 100 in Panels B7 and B8 in Fig 4). The LC3 (Panel B11 in Fig 4) and Lamp1 (Panel B12 in Fig 4) observed in fenestrated-spheroids (Panels B9 and B10 in Fig 4) showed co-localization inside the spheroid (Panel B13 in Fig 4), suggesting the production of autophagolysosomes in the fenestrated-spheroids. The findings of TH-LC3 (Panel B14 in Fig 4) and TH-Lamp1 (Panel B15 in Fig 4) co-localization suggest that DAergic debris are being stored in autophagolysosomes.

The finding of autophagosomes (LC3), lysosomes (Lamp1 and Lamp2), and autophagolysosomes (Lamp1/LC3 co-localization) in fenestrated-spheroids suggests that the autophagy process truncated in free-spheroids is activated in fenestrated-spheroids after the arrival of astrocytic processes.

### Astrocytes as the final destination for DAergic debris

**Fig 5:**
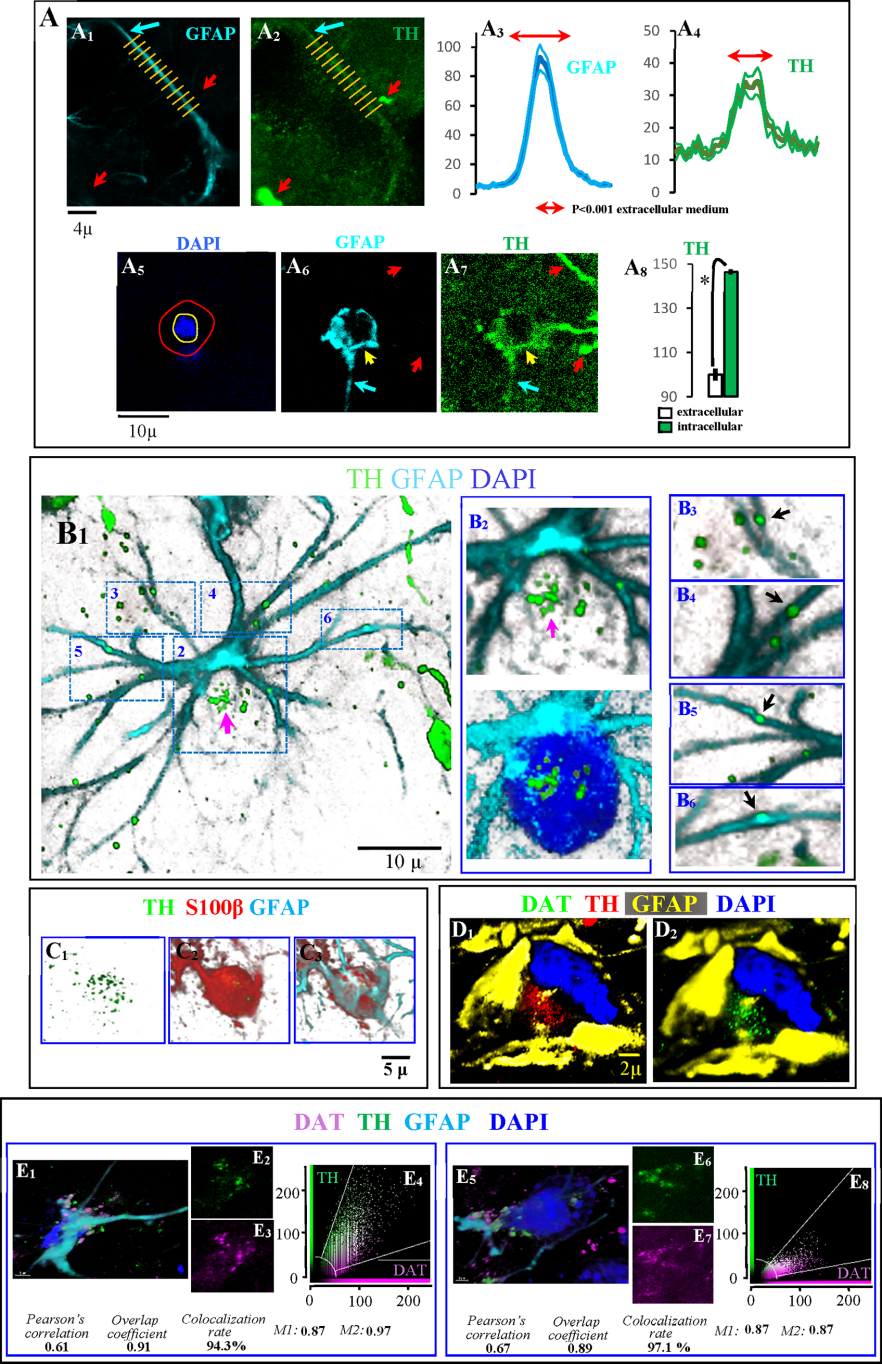
Dopaminergic protein within astrocytes. A1 shows an astrocytic process (blue arrow) which showed TH immunoreactivity (A2). Red arrows In A1 and A2 show free-spheroids without GFAP immunoreactivity. A3 and A4 show the distribution of GFAP and TH immunoreactivity across the yellow lines shown in A1 and A2 (mean ± standard error of yellow lines). A high TH immunoreactivity was also found in the cell soma of astrocytes. A6 shows an example of an astrocyte soma (yellow arrow) with TH immunoreactivity (A7). A8 (mean ± standard error) shows the TH immunoreactivity in the astrocyte soma (computed in the area between the cell nucleus -surrounded in fig A5 with a yellow circle- and the cell membrane -indicated with a red circle around the GFAP+ area of the astrocyte). In figs A6 and A7, red arrows show free-spheroids or DAergic axons without GFAP immunoreactivity, and blue arrows show astrocytic processes with TH immunoreactivity. B: an example of an astrocyte (B1) which showed TH immunoreactivity in its processes (black arrows in B3-B6) but also in its soma (B2). C1-C3 show an example of TH immunoreactivity (C1) in the soma of an astrocyte which presents S100β immunoreactivity (generally observed in cytoplasmic regions near the nucleus of astrocytes; C2) surrounded by GFAP immunoreactivity (generally observed in peripheral regions of the astrocyte soma; C3). DAT immunoreactivity (green in D2) was found in the cytoplasmic regions of astrocytes near the nucleus (blue) and which also showed TH immunoreactivity (D1). The TH and DAT immunoreactivity was observed in cytoplasmic regions of astrocytes near the nucleus (blue) and between the GFAP+ intermediate filaments which are normally located in more peripheral regions of the cytoplasm (yellow). Figs E1-E3 and E5-E7 show two examples of astrocytes showing TH (E2 and E6) and DAT (E3 and E7) immunoreactivity in the soma of astrocytes (E1 and E5). Both showed a marked subcellular co-localization of TH and DAT (E4 and E8; M1 is the Manders overlap coefficient which indicates the fraction of the marker 1 overlapping with marker 2, and M2 is that of marker 2 overlapping with marker 1 respectively).

Some days after the 6OHDA administration, different proteins which are characteristic of DA-ergic neurons were identified in astrocytes (Fig 5). Astrocytic processes (Panel A1 in Fig 5) showed immunoreactivity for TH (Panel A2 in Fig 5). In the above figures, the blue arrow shows an astrocytic process with both GFAP and TH immunoreactivity, and red arrows show two free-spheroids with TH but not with GFAP immunoreactivity. This fact was verified when the GFAP (Panel A3 in Fig 5) and TH (Panel A4 in Fig 5) distributions (mean ± standard error of values obtained in the position of the yellow lines in the Panels A1 and A2 in the Fig 5 example) were computed. High TH immunoreactivity was also found in the cell soma of astrocytes (Panel A8 in Fig 5). Panels A5-A7 in Fig 5 show an example of the high TH-immunoreactivity found in the cell soma of an astrocyte (computed in the area between the cell nucleus -surrounded here with a yellow circle- and the cell membrane -indicated with a red circle around the GFAP+ area of the astrocyte). TH immunoreactivity was often found grouped in the processes (Panels B3-B6 in Fig 5) and soma (Panel B2 in Fig 5) of astrocytes (Panel B1 in Fig 5). Fig 5C1–5C3 show an example of TH+ vesicles in the soma of an astrocyte which presents S100β immunoreactivity (characteristic of the cytoplasmic regions near the nucleus) surrounded by GFAP immunoreactivity (peripheral regions of the astrocyte soma). Other DAergic markers such as DAT were also found in the astrocytic soma. D images in Fig 5 show TH (Panel D1 in Fig 5) and DAT (Panel D2 in Fig 5) immunoreactivity near the nucleus (blue) of an astrocyte and between GFAP+ intermediate filaments (yellow) which are normally located in more peripheral regions of the cytoplasm. Panels E1-E4 in Fig 5 and Panels E5-E8 in Fig 5 show two examples of DAT and TH immunoreactivity in the cell soma of astrocytes. Two examples of DAT-TH co-localization in the denervated striatum are shown in Panels E4 and E8 in Fig 5 (co-localization indexes are shown at the bottom of Panel E in Fig 5) (see also the S10 Animation where spheroids and the TH immunoreactivity in the cytoplasm of an astrocyte are shown in green and the GFAP of astrocytes is shown in blue).

**Fig 6:**
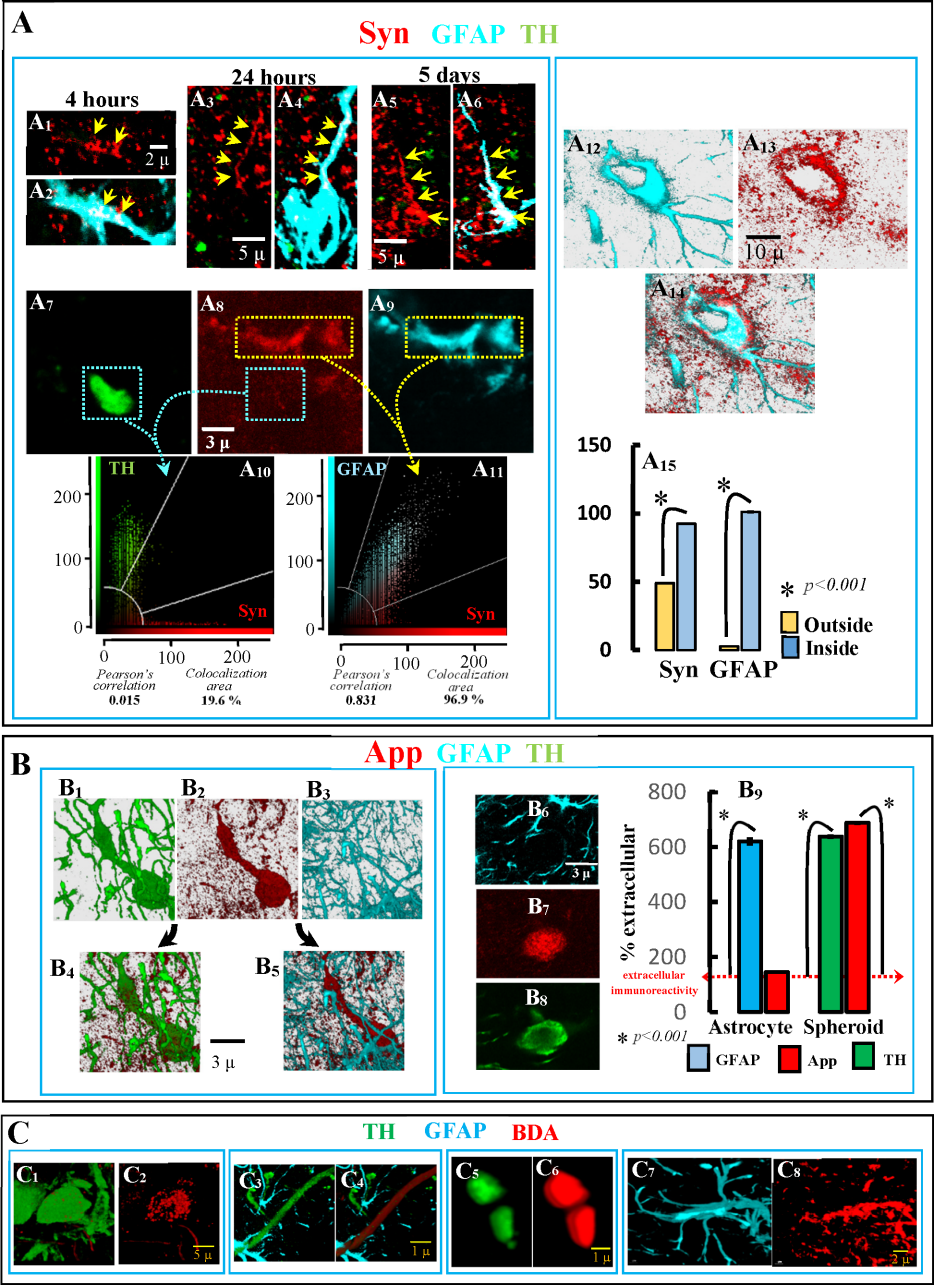
Syn, APP and BDA transfer to astrocytes. Syn was detected in astrocyte processes 4 hours (A1/A2), 24 hours (A3/A4) and five days (A5/A6) after 6OHDA administration, showing a Syn-GFAP colocalization (A8, A9 and A11) which was not observed in free-spheroids (A7, A8 and A10). Syn immunoreactivity was also found in the astrocyte soma (A12-A14). The astrocyte soma showed a high immunoreactivity level for GFAP and Syn (A15; values are mean ± standard error of values in the cytoplasm of astrocytes–inside- vs. those in the extracellular space–outside-). B4 and B5 show an example of the App accumulation (B2) in TH+ spheroids (B1) but not in GFAP+ astrocyte processes (B3). Another example of App accumulation in DAergic spheroids but not in astrocyte processes is shown in B6-B8. As shown in B9, App was found in DAergic spheroids but not in astrocytes (mean ± standard error of values normalized according to the extracellular immunoreactivity; n = 400 for astrocyte processes and n = 150 for spheroids). BDA injections in the posterior regions of the medial forebrain bundle filled DAergic cells of the nigrostriatal system (C1/C2 present the soma of a filled DA-ergic neurons and C3/C4 present a filled DAergic axon). BDA was found in striatal TH+ spheroids (C5/C6) of animals administered with BDA 30 days before the 6OHDA administration. C5 and C6 show an example of BDA incorporation into the processes of astrocytes of the DAergic-denervated striatum.

Syn, another synaptic protein not normally observed in astrocytes was also found in these cells a few hours after 6OHDA administration. Syn was detected in astrocyte processes 4 hours (Panels A1 and A2 in Fig 6), 24 hours (Panels A3 and A4 in Fig 6) and five days (Panels A5 and A6 in Fig 6) after 6OHDA administration. There was a Syn-GFAP co-localization in astrocyte processes (Panels A8, A9 and A11 in Fig 6), a fact not observed in free-spheroids (Panels A7, A8 and A10 in Fig 6). A Syn concentration was also found in the astrocyte somata (Panels A12-A14 in Fig 6), which showed a high immunoreactivity level for GFAP and Syn (Panel A15 in Fig 6). Unlike that observed for Syn (found in astrocytes but not in spheroids), App was found in free-spheroids (see Panels D1-D3 in Fig 1) but not in astrocytes (Panels B1-B5 and B6-B8 in Fig 6). Thus, the immunoreactivity of App was high in free-spheroids (vs. the extracellular level) but not in astrocytes (Panel B9 in Fig 6). Finally, BDA injections in the posterior regions of the medial forebrain bundle filled DAergic cells of the nigrostriatal system, including their cell soma (Panels C1 and C2 in Fig 6) and axon (Panels C3 and C4 in Fig 6). One month later, this tracing product was found in striatal TH+ spheroids generated by the ventricular administration of 6OHDA (Panels C5 and C6 in Fig 6), and in striatal astrocytic processes 4–6 days after 6OHDA (Panels C7 and C8 in Fig 6). This tracing study also supports the possibility that the cytosolic content of degenerating DAergic neurons is accumulated in spheroids and transferred to astrocytes.

As a whole, these data suggest that synaptic proteins of DAergic terminals may be transferred to astrocytes during the DA-ergic neuron degeneration, and that not all synaptic proteins follow the same transfer route.

### Cleaning DAergic debris

A summary of data obtained in free-spheroids, fenestrated-spheroids and astrocytes is shown in Table 2 where + means presence and—no detection of the protein. **Free-spheroids** showed immunoreactivity for TH/DAT (proteins of DA-ergic neurons), LC3 (autophagosome), and PS (“eat-me” signal), but not for GFAP and S100β (proteins of astrocytes), Stx17 (protein necessary for the fusion of autophagosomes to lysosomes) and Lamp1/Lamp2 (proteins of the lysosome). These data suggest that free-spheroids present the initial steps of autophagy but that these steps are not followed by the maturation of the autophagosome and the production of autophagolysosomes. Instead, an internalization of PS to autophagosomes could be produced in free-spheroids. **Fenestrated-spheroids** showed immunoreactivity for TH/DAT (showing their DAergic origin), GFAP/S100β (showing the astrocytic penetration), LC3 and Lamp1/Lamp2 (showing the presence of autophagosomes and lysosomes), and PS (“eat-me” signals). The finding of Lamp1/LC3 co-localization suggests the production of autophagolysosomes. Thus, the penetration of free-spheroids by astrocyte processes could both re-activate the processing of DAergic debris and open a window for the transfer of DAergic debris to the astrocytes. Processes and cell soma of astrocytes involved in the spheroid fenestration (GFAP/S100β) showed TH/DAT immunoreactivity, which suggests that the content of fenestrated-spheroids is transferred to astrocytes. App immunoreactivity was found in spheroids but not in astrocytes whereas Syn was found in astrocytes but not in spheroids. This suggests that these proteins of DAergic synapses are metabolized by different procedures, App could be directly metabolized in fenestrated-spheroids whereas Syn could be directly transferred to astrocytes and then metabolized in these cells. As a whole, present data suggest that astrocytes are involved in the metabolization of DAergic debris caused by the degeneration of synapses and axons of DA-ergic neurons.

**Table 2:** Response to the dopaminergic denervation of the striatum.

	Free-spheroid	Fenestrated-spheroid	Astocyte
TH	+	+	+
DAT	+	+	+
GFAP	-	+	+
S100β	-	+	+
GS	-	+	+
App	+	+	-
Syn	-	-	+
PS	+	+	
LC3	+	+	
Lamp1	-	+	+
Lamp2	-	-	+
Ubi	+	+	
Stx17	-	+	

TH: tyrosine hydroxylase, DAT: dopamine transporter, GFAP: glial fibrillary acidic protein, GS: glutamine synthetase, App: amyloid precursor protein, Syn: α-synuclein, App: amyloid precursor protein; PS: phosphatidylserine, Lamp1: lysosomal-associated membrane protein 1, Lamp2: lysosomal-associated membrane protein 2, Stx17: Syntaxin-17, Ubi: ubiquitin.

## Discussion

The present work was aimed at studying the metabolization of the striatal DAergic debris in an animal model of PD. It was observed that DAergic debris was grouped in spheroids and transferred to astrocytes which behaved as phagocytes. The degeneration of DAergic axons produced spheroids initially not associated to astrocytes (free-spheroids) which: 1. stored the debris of degenerating DA-ergic neurons (TH, DAT and App), 2. displayed partial autophagy (by producing autophagosomes without Stx17 and not linked to lysosomes) and started ubiquitination, and 3. apparently prepared their content for being phagocyted by other cells (adding PS to autophagosomes). Free-spheroids were penetrated by astrocytic processes (fenestrated-spheroids), which facilitated their processing by the lysosome. Finally, proteins initially identified in degenerating DAergic terminals were found in the processes and cell somata of astrocytes, suggesting that the cleaning of DAergic debris began within the damaged DA-ergic neurons and finished in the surrounding astrocytes. Taken together, present data suggest that this trans-cellular mechanism mixes a partial autophagy of DA-ergic neurons with an astrocytic phagocytosis (**transautophagy**).

As recently reported, the degeneration of the DAergic innervation of the striatum induced by injecting 6OHDA in the lateral ventricle is followed by a selective astrogliosis which is not accompanied by non-selective damage of the striatal tissue [23, 24]. This astrocytosis is similar to those observed in the parkinsonian striatum, including an upregulation of GFAP, GS, S100β, NDRG2 and vimentin but not of astrocytic proliferation or astrocytic scars [24]. In this study, microglia did not show the structural and neurochemical changes which characterize the triggering of phagocytic activity. The injection of 6OHDA in the striatal tissue induces macrophagic microgliosis, with microglial cells showing large amoeboid soma and CD68 immunoreactivity. These microglial cells also showed TH immunoreactivity, suggesting that they were engulfing dopaminergic debris around the injection site [23]. This fact was never found when 6OHDA was injected in the lateral ventricle, unless ventricle walls were directly damaged by the injecting needle [24]. However, these facts do not completely rule out any possible involvement of microglia in processing dopaminergic debris generated by the selective dopaminergic denervation of the striatum. This animal model was used here to study the role of astrocytes during the massive DAergic denervation which characterizes the first stages of PD.

The DAergic damage triggered an active reaction within the affected terminals in the striatum which transformed the unmyelinated thin axon (≈0.5μ diameter) and small synapses (≈1μ diameter) of DA-ergic neurons into massive structures which retained many of their components within a confined volume (end-bulb-like structures and spheroids with 2–9μ diameter). This storage of intracellular debris may prevent a jumbled dispersion of organelles and proteins of damaged cells across the extracellular space from hampering the normal interactions of undamaged cells, but, as present data suggest, this may also facilitate a fast withdrawal of debris.

The DAergic spheroids were initially not associated with astrocytes (free-spheroids). Free-spheroids showed many synaptic components of damaged DA-ergic neurons (TH, DAT and App) but not all of them. Syn is an example of a synaptic protein not accumulated by free-spheroids. Syn, normally present in DAergic synapses, is fibrillized and aggregated within DA-ergic neurons in PD, where it facilitates the production of axonal body neurites and somatic Lewy bodies [28, 29]. Syn can be released by exocytosis to the extracellular space [30, 31], where it is then absorbed via endocytosis by other cells including astrocytes [11, 13, 32, 33]. The high Syn immunoreactivity found in the extracellular space near the degenerating DAergic terminals and the early accumulation of Syn in astrocytic processes (at 4 hours after 6OHDA administration) suggest that this exocytosis/endocytosis route (which uses the extracellular space as a transient reservoir) is also at work in the present animal model. The Syn accumulation observed here in astrocytes was similar to that reported in the striatal astrocytes of PD patients [10, 34], and, as previously suggested, it could be useful to transport the protein to the blood via endothelial cells [11, 32, 33]. In any case, what present data show is that Syn degradation is different to that followed by other synaptic proteins and it does not need the direct contact between spheroids and astrocytes used by other proteins.

Different data suggested that the proteins retained within free-spheroids began to be processed in these structures. The immunohistochemical detection of ubiquitin is commonly used to detect ubiquitination of protein accumulations in different cell compartments, with the polyclonal anti-ubiquitin antibodies (which present a high affinity for ubiquitin-protein conjugates) being the most frequently used in such applications [35–37]. The polyclonal antibody used here showed a marked ubiquitin immunoreactivity in free-spheroids, suggesting that these structures present protein aggregates that are being ubiquitinated. On the other hand, the finding of clusters of LC3 immunoreactivity suggests the activation of autophagy in free-spheroids. During the formation of autophagosomes, the cytosolic precursor of LC3 is metabolized to form LC3-I which is then covalently conjugated to phosphatidylethanolamine to form LC3-II. LC3-II is specifically targeted to nascent autophagosomes, remaining tightly attached to the autophagosome membrane, where it is trapped and not exchanged with the cytoplasmic pools or with other vesicular structures. LC3-II could be the better marker to label autophagosomes [38, 39] but there are not selective antibodies for LC3-II, and LC3 antibodies recognize both the LC3-I of the cytosol and LC3-II of autophagosomes. Thus, the production of autophagosomes normally involves both an increase in LC3 levels and an accumulation of LC3 immunoreactivity in small cytosolic aggregates which is generated by the fixation of LC3-II to nascent autophagosomes [37, 38, 40–44]. This accumulation may be observed as fluorescent puncta in low magnification images of cells producing autophagosomes. However, when autophagosomes are close together in small volumes (such as those of spheroids) the identification of these structures needs high-magnification images. Present confocal images showed both an increase of LC3 level and vesicular aggregations of LC3 immunoreactivity in free-spheroids which suggest that these structures may produce autophagosomes, the first step of autophagy.

Autophagosomes generated at the beginning of autophagy need to mature before they link to lysosomes to form autophagolysosomes where intracellular debris may be finally metabolized. Stx17 is not normally found in phagophores and early autophagosomes, because it is only accumulated by mature autophagosomes where it facilitates their fusion to lysosomes [41]. Stx17 immunoreactivity was not found in free-spheroids suggesting that autophagosomes of these structures are not ready to be fused to lysosomes. In addition, neither Lamp1/Lamp2 (markers of lysosomes) immunoreactivity nor LC3/Lamp1-Lamp2 co-localization were found in free-spheroids, thus suggesting that these structures do not produce autophagolysosomes. These data suggest that the autophagy process which starts in free-spheroids is incomplete and does not conclude the debris metabolization. This should not be surprising because the autophagic process needs energy resources which are probably unavailable in free-spheroids, and because the final products of autophagy (amino acids, lipids…) are useless for a structure which, as occurs with spheroids, is heading for disappearance.

If DAergic debris cannot be entirely metabolized in free-spheriods then there should be some alternative process suitable to complete the debris degradation. Thus, the authors hypothesized that the autophagy process which starts in free-spheroids is concluded in the surrounding cells. PS is a phospholipid normally present in the internal side of cell membranes and which is externalized to the extracellular side of the membrane in damaged cells which are being prepared for phagocytosis (it behaves as an “eat-me” signal) [45–47]. No evidence of PS externalization was found in free-spheroids. Instead, the phospholipid was transferred from the cell membrane to internal regions of the spheroids where it co-localized with LC3. It has been recently reported that PS may be transferred to autophagosome-like organelles (LC3-II+) which are being targeted for transfer to other cells and not for fusion with lysosomes [48]. Thus, the PS internalization and LC3/PS co-localization found in free-spheroids suggest the preparation of the internal components of free-spheroids (more than the free-spheroids as a whole) for an external phagocytosis.

The reduction in the distance between free-spheroids and astrocytes observed during the DAergic degeneration suggested astrocytes as candidates to phagocyte the internal components of spheroids and to conclude the processing of DAergic debris. Free-spheroids are too big to passively diffuse across the extracellular space (as subcellular structures such as exosomes or ectosomes can), and they probably do not have the mechanisms and the energy resources needed to actively move themselves across the striatal tissue. Thus, the proximity of the free-spheroids and the astrocyte surface observed here the days after starting the DAergic degeneration was probably the result of the approach of the astrocytic process. Free-spheroids were surrounded by astrocytic processes which often crossed the spheroids several times from one side to the other (fenestrated-spheroid). Fenestrated-spheroids showed proteins which were not found in free-spheroids and whose finding suggest that the arrival of astrocyte processes to spheroids may trigger a second step for the degradation of the DAergic debris. Fenestrated-spheroids showed Lamp1 and Lamp2 immunoreactivity, a fact not observed in free-spheroids and which suggests the presence of lysosomes. The LC3-Lamp1 co-localization observed in fenestrated-spheroids suggests the production of autophagolysosomes in these structures. The finding of LC3-TH and Lamp1-TH co-localizations suggests that these autophagolysosomes contain the DAergic debris previously stored in free-spheriods. Taken together, these data suggest that the processing of DAergic debris which started in free-spheroids is activated after the penetration of these structures by astrocytic processes and the generation of fenestrated-spheroids.

DAergic proteins not normally observed in astrocytes (TH, DAT) were found in the cytoplasm of astrocytes of the denervated striatum. A similar accumulation was observed for Syn, despite the fact that the transfer of this protein to astrocytes did not involve spheroids. All these proteins were initially found in astrocytic processes and later in the cytosolic regions near the astrocyte nucleus. These data suggest that proteins of degenerating DA-ergic neurons are transferred to the astrocytic processes and then moved to the astrocytic soma. A similar transmission of DA-ergic debris to astrocytes could occur for non-protein contents of spheroids, a fact suggested by the finding of DBA in striatal astrocytes. This neuroanatomical tracer injected in the posterior region of the medial forebrain bundle was taken up by DA-ergic neurons (it was observed in the axon and soma of these cells), and was found, after the administration of 6OHDA, in DAergic spheroids. BDA was also observed in astrocytic processes of the denervated striatum, suggesting that non-protein contents of DAergic spheroids may also be transferred to astrocytes.

In summary, present data provide evidence that striatal debris originated by the DAergic degeneration is processed by a complex mechanism which involves the cooperation of the degenerating DAergic terminals and the surrounding astrocytes (transautophagy). Even in striatal regions subjected to a full DAergic degeneration, transautophagy could be efficient enough to clean the striatal tissue from a massive DAergic degeneration in a few days [23, 24], although the possible participation of other phagocytes cannot be ruled out at the moment. Transautophagy is a complex mechanism which includes: 1. a grouping of DAergic debris within free-spheroids; 2. an arrangement of the intra-spheroidal debris within autophagosomes (LC3); 3. a preparation of autophagosomes for their phagocytosis by other cells (PS); 4. a coming together of astrocytic processes which finally penetrate spheroids (fenestrated-spheroids); 5. a re-activation of the debris processing in fenestrated-spheroids (autophagolysosomes); 6. a transfer of the internal components of fenestrated-spheroids to the cytoplasm of astrocytes; 7. transport of DAergic “imported” proteins (TH, DAT) to the cell soma of astrocytes were they may continue their degradation. Transautophagy is probably not the only mechanism used by astrocytes to clean DAergic debris, as suggested by the finding of an extracellular-mediated transfer of Syn from degenerating DA-ergic neurons to astrocytes.

The actual significance of transautophagy in PD needs further studies in the human brain. Different evidence suggests that the physiological reconfiguration of the axons and synapses of DA-ergic neurons in the striatum begins to fail before the first motor signs of PD are detected, thereby generating an abnormal axonal trafficking of proteins and mitochondria, and an accumulation of proteins (e.g., α-synuclein) and organelle cargos in axonal spheroids. Present data suggest that if astrocytes were working correctly before the onset of PD, the transautophagy should be efficient enough to metabolize all DAergic debris. If this is not the case, the activation of microglia could be necessary to complete the metabolization of DAergic debris, and this would probably accelerate the onset and progression of PD. A good collaboration between degenerating DA-ergic neurons and astrocytes may facilitate a fast and quiet removal of DAergic debris without needing the potentially dangerous activation of “professional” phagocytes. Therefore, a failure of transautophagy (e.g., induced by the ageing of astrocytes) [2, 49] could accelerate the onset and development of PD.

## Conclusions

Evidence is reported here showing that debris generated by the DAergic denervation of the striatum is grouped in spheroids which produce autophagosomes with a high-level of PS. These spheroids are penetrated by astrocytic processes which facilitate the presence of lysosomes and the formation of autophagosomes. Finally, the DAergic debris is observed in the processes and soma of astrocytes. These data suggest that the metabolization of neuronal debris generated by the DAergic denervation of the striatum is generated by a complex process that starts in DAergic spheroids, progresses after the astrocytic fenestration of these spheroids and finishes in the astrocyte soma. This transautophagy is probably not the only mechanism for cleaning the DAergic debris, as suggested by the different dynamics shown by other proteins of the degenerating DAergic neurons (App and Syn).

## Supporting information

S1 Animation A free-spheroid (TH in green) near an astrocyte (GFAP in cyan). (MP4)

S2 Animation Aggregation of ubiquitin (in violet) in a DAergic spheroid (TH in green). (MP4)

S3 Animation A fenestrated-spheroid (TH in green and GFAP in cyan) which in S4 Animation presents internal aggregations of phosphatidylserine. (MP4)

S4 Animation Aggregations of phosphatidylserine (in red) in the fenestrated-spheroid shown in S3 Animation. (AVI)

S5 Animation A fenestrated-spheroid (TH in green) penetrated by an astrocytic process (GFAP in red). (AVI)

S6 Animation Fenestrated-spheroid which, as shown in S7 Animation, was penetrated by an astrocytic process (TH in green). (MP4)

S7 Animation Astrocytic process which penetrated the spheroid shown in S6 Animation (GFAP in cyan). (MP4)

S8 Animation Fenestrated-spheroid shown in S6 and S7 Animations, and which presents Lamp1 immunoreactivity (TH in green and Lamp1 in red). (MP4)

S9 Animation Co-localization of Lamp1 in the fenestrated-spheroid shown in S6, S7 and S8 animations (GFAP in cyan, TH in green and Lamp1 in red). (MP4)

S10 Animation Localization of TH immunoreactivity (in green) in a process of an astrocyte (GFAP in cyan). (MP4)

## Abbreviations

App: amyloid precursor protein
Casp6: activated Caspase 6
DA: dopamine
DA-ergic neurons: dopamine neurons
DAT: dopamine transporter
GFAP: Glial fibrillary acidic protein
GS: glutamine synthetase
Lamp1: lysosomal-associated membrane protein 1
Lamp2: lysosomal-associated membrane protein 2
NGS: normal goat serum
PD: Parkinson’s disease
PS: phosphatidylserine
SN: substantia nigra
Syn: α-synuclein
TH: tyrosine hydroxylase
6OHDA: 6-hydroxydopamine
